# Allopurinol Therapy in Gout Patients Does Not Associate with Beneficial Cardiovascular Outcomes: A Population-Based Matched-Cohort Study

**DOI:** 10.1371/journal.pone.0099102

**Published:** 2014-06-04

**Authors:** Victor C. Kok, Jorng-Tzong Horng, Wan-Shan Chang, Ya-Fang Hong, Tzu-Hao Chang

**Affiliations:** 1 Public Health and Clinical Informatics Research Group, Department of Biomedical Informatics, Asia University, Wufeng, Taichung, Taiwan; 2 Department of Internal Medicine, Kuang Tien General Hospital, Shalu, Taichung, Taiwan; 3 Department of Computer Science and Information Engineering, National Central University, Jhongli, Taoyuan, Taiwan; 4 Institute of Molecular Biology, Academia Sinica, Nankang, Taipei, Taiwan; 5 Graduate Institute of Biomedical Informatics, Taipei Medical University, Taipei, Taiwan; Johns Hopkins Bloomberg School of Public Health, United States of America

## Abstract

**Introduction:**

Previous studies have shown an association between gout and/or hyperuricemia and a subsequent increase in cardiovascular disease (CVD) outcomes. Allopurinol reduces vascular oxidative stress, ameliorates inflammatory state, improves endothelial function, and prevents atherosclerosis progression. Accordingly, we tested the hypothesis that a positive association between allopurinol therapy in gout patients and future cardiovascular outcomes is present using a population-based matched-cohort study design.

**Methods:**

Patients aged ≥40 years with newly diagnosed gout having no pre-existing severe form of CVD were separated into allopurinol (n = 2483) and non-allopurinol (n = 2483) groups after matching for age, gender, index date, diabetes mellitus, hypertension, hyperlipidemia, and atrial fibrillation. The two groups were also balanced in terms of uric acid nephrolithiasis, acute kidney injury, hepatitis, and Charlson comorbidity index.

**Results:**

With a median follow-up time of 5.25 years, the allopurinol group had a modest increase in cardiovascular risk [relative risk, 1.20; 95% confidence interval (CI), 1.08–1.34]. A Cox proportional hazard model adjusted for chronic kidney disease, uremia, and gastric ulcer gave a hazard ratio (HR) for cardiovascular outcomes of 1.25 (95% CI, 1.10–1.41) in gout patients receiving allopurinol compared with the non-allopurinol group. In further analysis of patients receiving urate-lowering therapy, the uricosuric agent group (n = 1713) had an adjusted HR of 0.83 (0.73–0.95) for cardiovascular events compared with the allopurinol group.

**Conclusions:**

The current population-based matched-cohort study did not support the association between allopurinol therapy in gout patients with normal risk for cardiovascular sequels and beneficial future cardiovascular outcomes. Several important risk factors for cardiovascular disease, such as smoking, alcohol consumption, body mass index, blood pressure were not obtainable in the current retrospective cohort study, thus could potentially bias the effect estimate.

## Introduction

Gout is a common disease resulting from urate crystal deposition in joints, connective tissues, and parenchymal organs including the kidneys, which may present clinically as gouty arthropathy, accumulation of urate crystals in the form of tophaceous deposits in numerous tissues, gouty nephropathy, or uric acid nephrolithiasis.[1] The prevalence of gout among the general United States population in 2007–2008 was 3.9% (8.3 million individuals).[2] All gout patients have hyperuricemia, defined as extracellular fluid urate supersaturation, at some point during the course of their illness. The prevalence of hyperuricemia is 10%–20% in the Western population.[1] Over the past two decades, a number of studies have shown that hyperuricemia portends an increased risk for subsequent cardiovascular outcomes, including mortality.[3]–[7]

In a population-based cohort study, we showed that among non-diabetic individuals aged ≥50 years who had no pre-existing serious cardiovascular disease (CVD), those with gout were 1.1 times more likely than those without gout to die from CVD in the next 5 years.[8]

The causative role of elevated serum uric acid concentrations (hyperuricemia) in CVD is suggested by studies showing its deleterious effect on endothelial function, inducing production of pro-inflammatory and pro-oxidative substances and platelet adhesiveness.[6]

There are three main types of urate-lowering therapy in clinical practice: reduction of urate production by use of a xanthine oxidase inhibitor such as allopurinol; enhancement of urinary excretion of uric acid with uricosuric agents; or conversion of uric acid to the more soluble purine end product allantoin using exogenous urate oxidase, also known as uricase. Allopurinol, as an antihyperuricemic agent, has been demonstrated to protect the cardiovascular system by reducing vascular oxidative stress, ameliorating inflammatory state, improving endothelial function, and preventing atherosclerosis progression.[4], [9]–[14] At higher doses (≥300 mg a day), allopurinol was even able to regress the left ventricular mass in patients with ischemic heart disease.[15]

The positive effect of allopurinol treatment on future cardiovascular risk among patients with chronic kidney disease (CKD) was shown in a small prospective randomized trial of 113 patients with estimated glomerular filtration rates of <60 milliliter/min. In this open label study conducted by Spanish investigators, patients were randomly assigned to treatment with allopurinol at 100 mg/day (n = 57) or to continue their usual therapy (n = 56). Allopurinol treatment slowed renal disease progression independently of age, gender, diabetes, C-reactive protein, albuminuria, or renin–angiotensin system blockers use. After a mean follow-up time of 23.4±7.8 months, allopurinol treatment reduced the risk of cardiovascular events by 71% [hazard ratio (HR), 0.29; 95% confidence interval (CI), 0.09–0.86] compared with the usual therapy.[16]

A cohort study using a record linkage database to study the impact of allopurinol treatment on cardiovascular and mortality outcomes showed that higher doses of allopurinol were associated with better control of urate and lower risks of both cardiovascular events and mortality. In this study, there was no significant increase in the risk of cardiovascular events for allopurinol users (n = 1035) when compared with non-urate-lowering therapy users [adjusted hazard ratio (aHR), 1.10; 95% CI, 0.95–1.26]. Compared with low-dose (100 mg) allopurinol users, high-dose (≥300 mg) users showed a significant reduction in the risk of cardiovascular events (aHR, 0.69; 95% CI, 0.50–0.94) and mortality (aHR, 0.75; 95% CI, 0.59–0.94).[17]

An observational study using data from a Health Maintenance Organization evaluated the effect of uric acid and allopurinol use on clinical outcomes in 6204 patients with heart failure. This study demonstrated that high uric acid levels (>7.7 mg/dL or >458 µmol/L) were a predictor of increased mortality (aHR, 1.37; 95% CI, 1.17–1.60) and an increase in cardiac hospitalizations (aHR, 1.10; 95% CI, 1.01–1.22). An increase in uric acid levels during follow-up was also an independent predictor of mortality (aHR, 1.46; 95% CI, 1.25–1.71) and cardiac hospitalizations (aHR, 1.15; 95% CI 1.06–1.23). With a median follow-up time of 498 days, treatment with allopurinol was independently associated with improved survival (aHR, 0.79; 95% CI, 0.64–0.98).[18] Another large, time-matched, nested case-control analysis using health care databases in Quebec, Canada, was designed to determine whether gout and allopurinol use are associated with heart failure outcomes. This study, which targeted a cohort of elderly patients aged ≥66 years with heart failure (n = 25,090), showed that >30 days of continuous allopurinol use is associated with reduced heart failure readmissions or death (HR, 0.69; 95% CI, 0.60–0.79; *P*<0.001) and all-cause mortality (HR, 0.74; (95% CI, 0.61–0.90; *P*<0.001) among patients with a history of gout.[19]

Therefore, it would be of great interest and academic appeal to examine the hypothesis that among different urate-lowering therapies for gout patients, allopurinol therapy would have better cardiovascular outcomes than non-allopurinol treatments, including a uricosuric agent in matched patients with gout. We thus designed a large-scale population-based retrospective matched-cohort study to answer this question.

## Methods

### Data Sources

We conducted a population-based retrospective matched-cohort study using the Taiwan National Health Insurance Research Database (NHIRD). The Taiwan National Health Insurance (NHI) program commenced on March 1, 1995. As of 2007, 98.4% of Taiwan's population of 22.96 million individuals was enrolled in this program. The NHIRD contains a number of computerized databases that incorporate files and original data on claims reimbursements derived from the insurance system held by the NHI Administration. These data have been provided to researchers for academic research purposes. The NHIRD comprises four main database files: ambulatory expenditures by visit, inpatient expenditures by admission, details of ambulatory care orders, and details of inpatient orders. These data files were de-identified by scrambling the identification codes of both individuals and medical facilities. The NHIRD is widely regarded as very accurate and complete and has become one of the largest and most comprehensive population-based databases in the world.[8], [20]–[22]

This study used a dataset of one million randomly selected enrollees representing 4.5% of the Taiwanese population in the entire NHI enrollee profile. There was no significant difference in age or gender between the patients in the dataset used in this study and the population in the mother NHIRD, as verified by the National Health Research Institute (NHRI), Taiwan.

### Ethics Statement

Because this study used only NHIRD data files that were de-identified by scrambling the identification codes of both individuals and medical facilities, this research fits the criteria for exemption from review by the Institution Review Board contained within the legal statements promulgated by the Ministry of Health and Welfare of Taiwan pursuant to Paragraph 1, Article 5 of the *Human Subjects Research Act* enacted on December 28, 2011. This study adhered to strict confidentiality guidelines that are in accordance with the regulations set forth by the *Personal Information Protection Act* of Taiwan, amended on May 26, 2010. The research was conducted in accordance with the *Declaration of Helsinki* as revised in 1989.

### Study Population and Study Cohorts

We limited our study population to every individual diagnosed with gout in the years 1999 to 2008 (n = 46,586). We defined individuals with gout as those with gout-related diagnoses according to code 274 of the International Classification of Diseases, 9^th^ Revision, Clinical Modification (ICD-9-CM). Under ICD-9-CM code 274, subjects with gouty arthropathy (code 274.0), gouty nephropathy (274.1), gouty iritis (274.89), gouty tophi (274.8), gouty tophi of ear (274.81), gouty tophi of heart (274.82), gout with other manifestations (274.89), and uric acid nephrolithiasis (274.11) were included. Gout and gout-related diagnoses are used interchangeably in this paper.

**Figure 1:**
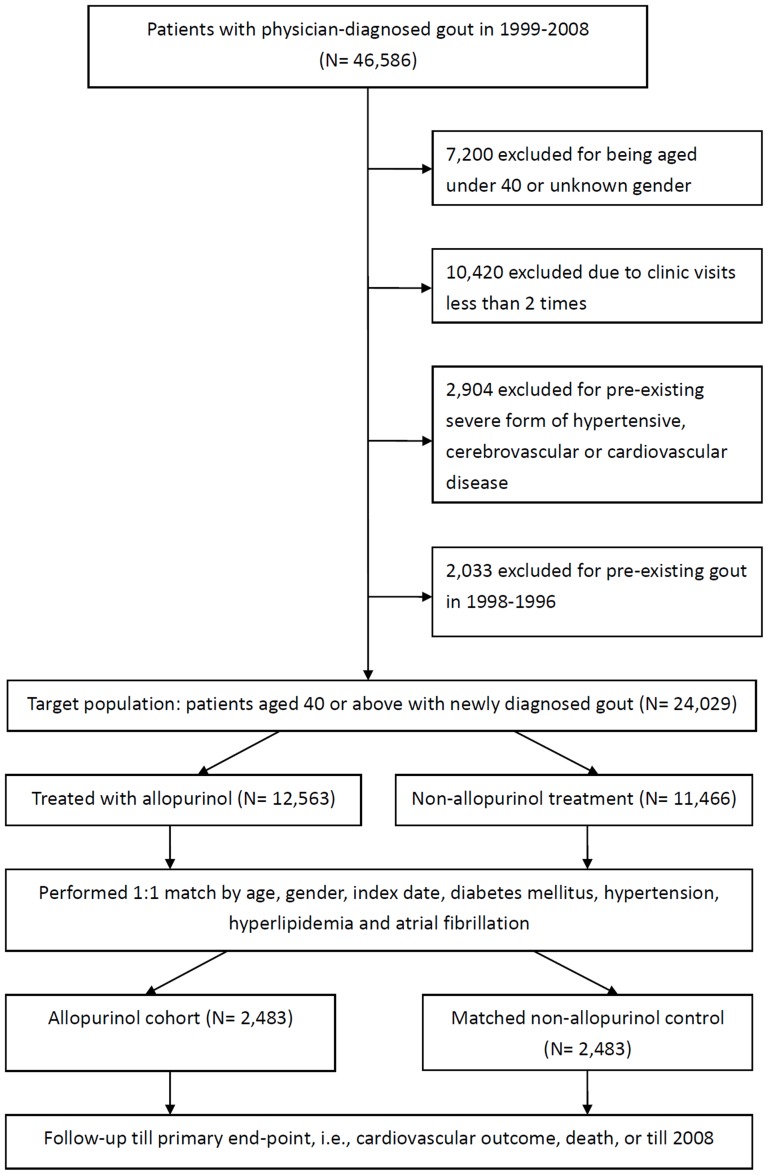
Study flowchart showing target population, exclusion reasons, matching and follow-up details in this retrospective matched-cohort population-based study using Taiwan National Health Research Institute Database.

From this pool of 46,586 subjects with gout, we excluded 7200 subjects who were <40 years old or whose gender was unknown; 10,420 who visited the clinic less than 2 times during the defined study period; 2904 who had a pre-existing severe form of hypertensive, cerebrovascular, or CVD; and 2033 who had pre-existing gout in the preceding 2 years. This process resulted in a target population of 24,029 patients aged ≥40 years, with newly diagnosed gout, having no history of pre-existing severe form of hypertension, cardiovascular, or cerebrovascular disease (Figure 1).

The severe CVDs included malignant hypertension, hypertensive nephropathy, myocardial infarction, or any form of congestive heart failure, as well as a coronary artery bypass surgery or percutaneous transluminal coronary angioplasty. In addition, subjects with intracerebral hemorrhage, intracranial hemorrhage, occlusion/stenosis of pre-cerebral arteries, and occlusion of cerebral arteries were also excluded. The detailed list of the corresponding ICD-9-CM codes can be found in our previously published article.[8] By excluding this group of patients, we ensured that the study would not contain patients with pre-existing severe cardiovascular and/or cerebrovascular comorbidities and, therefore, with a high pre-existing risk of a cardiovascular outcome.

The study population was divided into two groups: those who received allopurinol treatment and those who did not. The allopurinol group consisted of 12,563 patients and the non-allopurinol group consisted of 11,466 patients. We then performed one-to-one matching by age at accrual, gender, index date of subjects in the allopurinol group, diabetes mellitus, hypertension, hyperlipidemia, and atrial fibrillation. After matching, we finalized two matched study group, an allopurinol group and a non-allopurinol group, each containing 2483 patients. Follow-up continued until the occurrence of a primary cardiovascular outcome, death, or the end of 2008 (Figure 1).

### Main Outcome Measures

A cardiovascular outcome is defined as an event requiring hospitalization during follow-up that is given a primary final diagnosis among several CVD-related diagnoses. These diagnoses included a spectrum of diseases from coronary heart disease (CHD) (ICD-9-CM codes 410–414) to hypertensive heart disease (401–405), heart failure (428), cerebrovascular disease (stroke) (430–438), and other CVDs (390–400, 406–409, 415–427, 429, and 439–459).[8], [23] Cases with fatal CVD event ended up in the Emergency Room that did not require hospitalization were very rare in Taiwan due to practice culture and fairly easy access to hospitals.

Demographic data such as age and gender, as well as medical comorbidities including hypertension, diabetes mellitus, hyperlipidemia, atrial fibrillation, uric acid nephrolithiasis, acute kidney injury, hepatitis, contact dermatitis and other eczema, CKD, uremia, and gastric ulcer were collected for baseline evaluation. The Charlson comorbidity index (CCI) was also calculated for each case in the two groups. Comorbid medical conditions, identified using their standard ICD-9-CM codes, were used to calculate cumulatively the CCI score for each individual. The established CCI, adapted from the Charlson index for use with ICD-9-CM coded administrative databases, contains 17 weighted categories related to chronic concomitant diseases and is able to predict the subsequent 1-year mortality among inpatients. Each category has a score between 1 and 6 points (1 point each for myocardial infarction, congestive heart failure, peripheral vascular disease, cerebrovascular disease, dementia, chronic lung disease, rheumatologic disease, peptic ulcer disease, mild liver disease, and diabetes without organ damage; 2 points each for diabetes with organ damage, hemiplegia or paraplegia, severe renal disease, and any malignancy including leukemia and lymphoma; 3 points for severe liver disease; and 6 points each for metastatic solid tumor and HIV infection), and the sum of these scores is regarded as a measure of the burden of medical comorbidity.[24], [25]

We assessed the use of uricosuric agents for urate-lowering therapy in the comparator cohort not receiving allopurinol. In Taiwan, three kinds of uricosuric agent are reimbursed: benzbromarone, probenecid, and sulfinpyrazone. The daily dose and duration of allopurinol use were also evaluated in this matched-cohort study.

### Statistical Analysis

Pre-analysis data file merging and other data management operations before statistical analysis were performed using the SAS statistical package (SAS System for Windows, version 8.2, SAS Institute, Cary, NC, USA). Statistical analyses were performed using SPSS software (version 17.0, SPSS Inc., Chicago, Illinois, USA). All statistical tests were two-sided. Values of *P*<0.05 were considered statistically significant. The risk of cardiovascular outcomes associated with gout was evaluated using Cox proportional hazards analysis. All Cox regression models included the following covariates: CKD, uremia, and gastric ulcer. Adjusted hazard ratios (aHR) with a 95% CI were calculated. The relative risks of cardiovascular outcomes were calculated and analyzed using the Chi-square test (2×2). The distribution of comorbidity characteristics were compared between the two groups, and the differences were examined using the Chi-squared test and t test, as well as the Mann–Whitney U test (also called the Wilcoxon rank-sum test), which is a non-parametric statistical hypothesis test for assessing whether one of two samples of independent observations tends to have larger values than the other. Variables such as age, sex, underlying comorbidity, daily dose of allopurinol, use of uricosuric agents, duration of allopurinol use, and follow-up in years are reported as percentages. StatsDirect statistical software (StatsDirect Ltd, England, 2008) was used to provide relative risk values and Yate's corrected 2×2 test computations. The cardiovascular event-free cumulative survival probabilities were estimated in each group using the Kaplan–Meier method. The log-rank test was used to compare the significance of inequalities with respect to the cardiovascular event-free survival curves of each group.

## Results

Two matched groups, allopurinol and non-allopurinol, each consisting of 2483 patients, were followed-up throughout the defined study period, or until a cardiovascular event, with neither drop-out nor cross-over. Because the allopurinol and non-allopurinol groups were already matched with respect to seven pre-defined conditions (age at diagnosis of gout, gender, index date of the allopurinol group subjects, diabetes mellitus, hypertension, hyperlipidemia, and atrial fibrillation), there was no statistically significant difference between these two groups in terms of these seven variables. Furthermore, there was no statistically significant difference in the frequency of uric acid nephrolithiasis, acute kidney injury, hepatitis, contact dermatitis and other eczema, as well as overall Charlson–Deyo comorbidity index (Table 1). However, there was a statistically significant higher percentage of CKD and uremia in the allopurinol group compared with the non-allopurinol group (4.75% and 1.09% versus 2.38% and 0.52%, respectively). The allopurinol group also had a higher percentage of CKD, including uremia, although the percentage was considered low at approximately 7%. The allopurinol group had a slightly higher rate of gastric ulcer (2.34% versus 1.49%; *P* = 0.0296) (Table 1). These minor inequalities were later adjusted in the Cox proportional hazard model to calculate the HR.

**Table 1:** Baseline characteristics of the two matched cohorts of patients with newly diagnosed gout either treated with or without allopurinol.

Characteristics		Allopurinol cohort (n = 2483)		Non-allopurinol cohort (n = 2483)		*P*-value
		N	%	N	%	
Age at gout diagnosis						1.0000
	40–49	481	19.37	481	19.37	
	50–59	621	25.01	621	25.01	
	60–69	628	25.29	628	25.29	
	70–79	561	22.59	561	22.59	
	≥80	192	7.73	192	7.73	
	Mean ±SD	61.84±12.13		61.84±12.13		
Gender						1.0000
	Men	1545	62.22	1545	62.22	
	Women	938	37.78	938	37.78	
Co-morbidities						
	Hypertension	1257	50.62	1257	50.62	1.0000
	Diabetes mellitus	573	23.08	573	23.08	1.0000
	Hyperlipidemia	766	30.85	766	30.85	1.0000
	Atrial fibrillation	1	0.04	1	0.04	1.0000
	Uric acid nephrolithiasis	1	0.04	0	0.00	1.0000
	Acute kidney injury	9	0.36	3	0.12	0.0829
	Hepatitis	53	2.13	49	1.97	0.6890
	Contact dermatitis and other eczema	424	17.08	431	17.36	0.7925
	Chronic kidney disease	118	4.75	59	2.38	<.0001
	Uremia	27	1.09	13	0.52	0.0262
	Gastric ulcer	58	2.34	37	1.49	0.0296
	Charlson's Comorbidities Index (CCI)					0.3013*
	0	793	31.94	887	35.72	
	1	515	20.74	633	25.49	
	2	381	15.34	321	12.93	
	3	282	11.36	236	9.50	
	4	204	8.22	147	5.92	
	5	122	4.91	106	4.27	
	6	81	3.26	70	2.82	
	7	43	1.73	45	1.81	
	8	29	1.17	15	0.60	
	9	18	0.72	13	0.52	
	10	8	0.32	4	0.16	
	>10	7	0.28	6	0.24	

*Wilcoxon's signed rank test (2-sided P value).

The 2 cohorts were matched for age at gout diagnosis, gender, diabetes mellitus, hypertension, hyperlipidemia, atrial fibrillation and index date. In addition, both cohorts were balanced in terms of Charlson's comorbidities index (CCI), acute kidney injury and hepatitis etc.

The mean age (± standard deviation) of the study group was 61.84 (±12.13) years. Men accounted for 62.22% of the total cases. Slightly more than half of the patients in both groups had hypertension at accrual. Twenty three percent of the patients had been diagnosed with diabetes and approximately 31% had hyperlipidemia (Table 1).

In the allopurinol group, 85.54% of gout patients took allopurinol at a dose of >100 mg/day. Of these, 16% were treated with a dose of ≥300 mg/day. Among patients receiving allopurinol, 59% took allopurinol for >6 months. Of these, 9% took allopurinol for 0.5–1 year; 13% for 1–2 years; 9% for 2–3 years; 5% for 3–4 years and 23% for >4 years.

In the non-allopurinol group, nearly 69% of the patients received a uricosuric agent. The most common uricosuric agent used was benzbromarone (66%), followed by sulfinpyrazone (3%), and probenecid (0.4%).

Follow-up of the patients in the two groups was complete; there was no drop-out or loss to follow-up. The median follow-up time for the allopurinol group was 5.25 years [interquartile range (IQR), 4.88 years], whereas it was 5.04 years (IQR, 4.98 years) for the non-allopurinol group. There was no statistically significant difference in the follow-up time between these two groups (Table 2). There were 566 cardiovascular events (22.8%) in the allopurinol group during the entire follow-up period and 470 events (18.9%) in the non-allopurinol group. The relative risk for cardiovascular outcomes of the allopurinol group was 1.2 times higher than that of the non-allopurinol group (95% CI, 1.08–1.34) (Table 2). In another words, the allopurinol group was 1.2 times more likely to experience a cardiovascular outcome compared with the non-allopurinol group.

**Table 2:** Treatment with allopurinol by total daily dose, duration of use, duration of follow-up, and cardiovascular outcomes.

Treatment, follow-up, and outcomes		Allopurinol cohort (n = 2483)		Non-allopurinol cohort (n = 2483)		*P*-value
		N	%	N	%	
Allopurinol	daily dose	2483	100	-	-	
	<100 mg	359	14.46	-	-	
	100 mg	1262	50.83	-	-	
	200 mg	467	18.81	-	-	
	≥300 mg	395	15.90	-	-	
Uricosuric agent		-	-	1713	68.99	
	benzbromarone	-	-	1628	65.57	
	probenecid	-	-	11	0.44	
	sulfinpyrazone	-	-	74	2.98	
Duration of allopurinol use						
	<0.5 year	1011	40.72	NA	NA	
	0.5–1 year	233	9.38	NA	NA	
	1–2 years	325	13.09	NA	NA	
	2–3 years	215	8.66	NA	NA	
	3–4 years	134	5.40	NA	NA	
	>4 years	565	22.75	NA	NA	
Follow-up in years	Median, (IQR)	5.25, (4.88)		5.04, (4.98)		0.6971
***Cardiovascular outcome***	***Events***	566	22.80	470	18.93	
	***Relative Risk (RR)***	1.20 (95% CI = 1.08–1.34)				

With a median follow-up of 5.25 years, the allopurinol cohort has harbored a modestly increased risk of cardiovascular events.

CI: confidence interval; IQR: interquartile range; NA: not applicable; RR: relative risk; yr: year.

**Figure 2:**
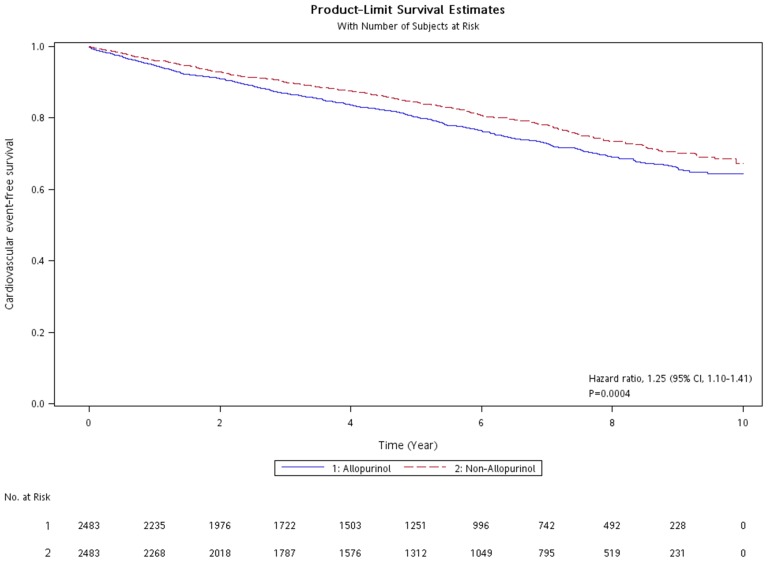
Kaplan-Meier cardiovascular event-free survival curves of gout patients treated with allopurinol and matched comparators of gout patients receiving no allopurinol. At a median follow up of 5.25 years, the allopurinol therapy group harbored an increased hazard ratio of 1.25 (95% confidence interval, 1.10–1.41).

A Cox proportional hazards regression model established to calculate the effect of allopurinol therapy on future cardiovascular outcomes for gout patients aged ≥40 years, after adjusting for CKD, uremia, and gastric ulcer gave an aHR of 1.25 (95% CI, 1.10–1.41) compared with the non-allopurinol therapy (Table 3 and Figure 2).

**Table 3:** Univariate and multivariate hazard ratios (HR) for cardiovascular outcome in patient with essentially newly diagnosed gout receiving allopurinol as compared with non-allopurinol medications.

Cardiovascular Outcome	N (%)	Univariate		Multivariate	
		HR	95% CI	HR	95% CI
Non-allopurinol	470 (18.93)	Ref	-	Ref	-
Allopurinol	566 (22.80)	1.24*	1.10–1.41	1.25**	1.10–1.41

Multivariate HR was obtained after adjusting for chronic kidney disease, uremia, and gastric ulcer.

*P<0.05;

**P<0.001.

CI: confidence interval; HR: hazard ratio; Ref: reference.

With respect to the different forms of cardiovascular events, we further examined the effect of allopurinol therapy on subsequent hospitalizations due to CHD, cerebrovascular disease (stroke), hypertensive heart disease, heart failure, or other cardiovascular disorders. The HR and aHR for hospitalization due to each form of CVD are reported in Table 4. Compared with non-allopurinol therapy, allopurinol therapy for patients aged >40 years with gout was significantly associated with an increased aHR for hospitalization due to CHD (aHR, 1.41; 95% CI, 1.10–1.79), hypertensive heart disease (aHR, 1.34; 95% CI, 1.18–1.53), and heart failure (aHR, 1.52; 95% CI, 1.17–1.98) after adjusting for uremia, CKD, and gastric ulcer.

**Table 4:** Hazard ratio and adjusted hazard ratio for hospitalization due to cardiovascular events in patients aged more than 40 having their gout treated with allopurinol as compared those matched control receiving no allopurinol.

	Allopurinol cohort (%) (n = 2,483)	Non-allopurinol control (%) (n = 2,483)	HR (95% CI)	Adjusted HR (95% CI)
Cardiovascular Events	566 (22.80)	470 (18.93)	1.24*** (1.10–1.41)	1.25*** (1.10–1.41)
Coronary heart disease	158 (6.36)	111 (4.47)	1.44** (1.13–1.84)	1.41** (1.10–1.79)
Cerebrovascular disease (Stroke)	176 (7.09)	150 (6.04)	1.18 (0.95–1.47)	1.18 (0.95–1.47)
Hypertensive heart disease	538 (21.67)	419 (16.87)	1.34*** (1.18–1.52)	1.34*** (1.18–1.53)
Heart failure	141 (5.68)	92 (3.71)	1.55** (1.19–2.02)	1.52** (1.17–1.98)
Other	233 (9.38)	208 (8.38)	1.13 (0.94–1.36)	1.12 (0.93–1.35)

Adjustment was made for uremia, chronic kidney disease and gastric ulcer.

*P<0.05,

**<0.01,

***<0.001.

CI: confidence interval; HR: hazard ratio.

We fit a Cox model for gout patients receiving urate-lowering therapy to determine if allopurinol provided protection against cardiovascular outcomes compared with uricosuric therapy consisting of benzbromarone, sulfinpyrazone, and probenecid. The results showed that when allopurinol therapy was regarded as a reference, uricosuric therapy was shown to have cardiovascular protection (aHR, 0.83; 95% CI, 0.73–0.95) (Table 5).

**Table 5:** Hazard ratio and adjusted hazard ratio for hospitalization due to cardiovascular events in patients or matched subjects aged more than 40 with gout and hyperuricemia receiving urate-lowering therapy, either allopurinol or uricosuric agent.

Urate-lowering therapy	Cardiovascular Events	Univariate Cox model		Adjusted Cox model	
	N (%)	HR	95% CI	HR	95% CI
Allopurinol (n = 2483)	566 (22.80)	ref	ref	ref	ref
Uricosuric agents (n = 1713)	334 (13.45)	0.83**	0.72–0.95	0.83**	0.73–0.95

Adjustment was made for uremia, chronic kidney disease and gastric ulcer.

*P<0.05,

**<0.01,

***<0.001.

CI: confidence interval; HR: hazard ratio

Taking all these results into account, allopurinol therapy did not provide cardiovascular protection and also led to adverse outcomes in patients with gout. With a median follow-up time of 5.25 years, the allopurinol group had a modest increase in cardiovascular risk (relative risk, 1.20; 95% CI, 1.08–1.34). A Cox proportional hazard model adjusted for CKD, uremia, and gastric ulcer gave an HR for cardiovascular outcomes of 1.25 (1.10–1.41) in gout patients receiving allopurinol compared with those not receiving allopurinol. In a subgroup analysis of patients receiving urate-lowering therapy, the uricosuric agent group (n = 1713) had an aHR of 0.83 (0.73–0.95) compared with the allopurinol group.

We also fit a Cox model examining the associations of low- and high-dose allopurinol therapy with subsequent cardiovascular risk. The model used a daily allopurinol dose of <100 mg, which had an incidence of cardiovascular events of 23.68%, as its reference for comparison (Table 6). Using this model, the incidence of cardiovascular events was 25.67%, 19.70%, and 11.48% in the 100-mg group, 200-mg group, and ≥300-mg group, respectively. The univariate HRs were statistically significant at the higher doses of 200 mg (HR, 0.73; 95% CI, 0.55–0.98) and 300 mg (HR, 0.67; 95% CI, 0.48–0.92), demonstrating the beneficial effect of a higher daily dose of allopurinol therapy. Although the 200-mg and ≥300-mg groups retain their beneficial HRs (both HR were 0.91) after adjustment for CKD, uremia, and gastric ulcer, the differences lose statistically significance (Table 6). Nevertheless, because of the comparatively lower numbers of cases in the higher dosage groups, a true benefit from a higher daily dose of allopurinol cannot be completely ruled out.

**Table 6:** Univariate and multivariate hazard ratios (HR) for cardiovascular outcome in patient with gout receiving higher dosage of allopurinol as compared with low-dose allopurinol.

Allopurinol therapy	Cardiovascular events	Univariate		Multivariate	
Daily dosage	Number (%)	HR	95% CI	HR	95% CI
<100 mg	85 (23.68)	Ref	-	Ref	-
100 mg	324 (25.67)	0.988	0.778–1.255	1.191	0.931–1.523
200 mg	92 (19.70)	0.731*	0.545–0.982	0.906	0.671–1.224
≥300 mg	65 (11.48)	0.669*	0.484–0.924	0.907	0.650–1.266

Multivariate HR was obtained after adjusting for chronic kidney disease, uremia, and gastric ulcer.

*P<0.05.

CI: confidence interval; HR: hazard ratio; Ref: reference.

## Discussion

To the best of our knowledge, this is the first population-based study accruing a large number of patients with gout and including well-matched references that was designed to uncover an association between allopurinol therapy and subsequent cardiovascular risk. The quasi-experimental design of the study, with clear exclusion criteria and extensive matching, allowed profound control of variance by exclusion of subjects with pre-existing severe CVDs and control of known confounding variables. Excluding subjects with pre-existing severe CVD results in the exclusion of high-risk to very-high risk patients who are prone to experience a cardiovascular outcome during subsequent follow-up. The extensive matching process also allowed, in the early phase of the study, elimination of confounding variables other than age, gender, and index date only; thus, post-hoc statistical adjustments could be omitted.

Selection bias was obviously avoided. The vast majority (84.5%) of the entire cohort received urate-lowering therapy (including allopurinol or uricosuric agents), indicating that selection methodology for the gout patients was adequate. Allocation bias was also avoided, because positive or negative exposure to allopurinol was unambiguous in the database. Furthermore, our use of administrative-based data enabled us to study allopurinol exposure and cardiovascular outcomes without the influence of recall bias in both groups. There was no drop-out or loss to follow-up because complete tracking of study subjects was feasible, even when a patient relocated or received treatment in different hospitals. The follow-up time was considered long enough to allow a cardiovascular event to be observed.

Using this study design, the results showed an association between allopurinol therapy for gout patients and increased CVD events. Allopurinol may have a harmful effect, with an increase in the risk of CVD even after adjustment for CKD and uremia. This means that a Taiwanese patient aged ≥40 years with newly diagnosed gout and hyperuricemia who is prescribed allopurinol therapy to lower urate levels is associated with a 25% increase in cardiovascular risk over the next 10 years of follow-up. This data is considered robust, with a narrow 95% confidence interval.

It is noteworthy that a published cohort study, using a United Kingdom record linkage database, conducted in a population of patients aged ≥60 years who underwent urate measurements either at a hospital or a community clinic, demonstrated that there was a statistically insignificant increase in the risk of cardiovascular events for allopurinol users compared with subjects not receiving urate-lowering therapy (aHR, 1.10; 95% CI 0.95–1.26) and with subjects not receiving urate-lowering therapy that had urate >6 mg/dL (aHR, 1.07; 95% CI, 0.89–1.28). Nevertheless, the study showed that compared with subjects receiving low-dose (100 mg) allopurinol, those receiving high-dose (≥300 mg) allopurinol showed a significant reduction in the risk of cardiovascular events (aHR, 0.69; 95% CI, 0.50–0.94).[17] Our analysis of HRs by different daily dosages of allopurinol (Table 6) also suggested that a higher daily dose may benefit patients with gout in terms of cardiovascular risk.

Another retrospective cohort study of patients with chronic heart failure that investigated the association between different durations (short-term versus long-term) and daily dosages [low-dose versus high-dose (≥300 mg)] of allopurinol therapy and cardiovascular mortality merits mention here.[26] The results of this study disclosed that long-term low-dose allopurinol worsened cardiovascular mortality, with HR of 2.04 (95% CI, 1.48–2.81); whereas, long-term high-dose allopurinol provided benefit, with a relative risk of 0.59 (95% CI, 0.37–0.95).

Thus, we believe that it is the dosage of allopurinol may make a difference when reduction of cardiovascular risk is the main therapeutic aim. Further prospective studies are needed to test this hypothesis.

The mechanisms underlying the beneficial effect of higher daily dosage of allopurinol have been reported in recent years. Erdogan et al. published a single arm clinical trial of allopurinol at 300 mg/day for 3 months in patients diagnosed with dilated cardiomyopathy with concomitant hyperuricemia. Their results showed that this high dose of allopurinol can improve coronary microvascular and left ventricular function.[9] Rajendra et al. reported a double-blind, randomized, placebo-controlled trial of very high-dose allopurinol therapy (600 mg/day) with cross-over in patients with coronary artery disease. Their results showed that at this very high dose, allopurinol profoundly reduces vascular tissue oxidative stress and improves three different measures of vascular/endothelial dysfunction.[27] Recently, Noman et al. conducted a double-blind, randomized, placebo-controlled trial of allopurinol at 600 mg/day for 6 weeks before cross-over in patients with at least 2 months of angiographically documented stable chronic angina pectoris with positive exercise tolerance test. They found that this very high-dose allopurinol could increase the median time to ST depression, median total exercise time, and time to chest pain.[28]

There is also a possibility that our result, that allopurinol fared worse than the matched reference cohort consisting of uricosuric agent users (69%) and patients having no urate lowering therapy (31%), may have resulted just because of the capability of allopurinol therapy to serve as a prognostic factor suggesting a poorer cardiovascular outcome. However, this suggestion is only hypothetical.

There are some potential limitations to this study. We did not have access to the relevant biochemical datasets incorporating urate measurements and personal lifestyle histories such as cigarette smoking and alcohol consumption history, abdominal obesity, consumption of fruits and vegetables, and records of physical activity. This is because individual identities are not available because of de-identification of the cases within the NHI databases. The lack of such information could potentially bias the effect estimate. Another potential limitation is that data for medication other than urate-lowering medications and the compliance and adherence to treatment were too complex to incorporate in the covariate analyses.

In conclusion, allopurinol therapy as a urate-lowering treatment has previously been found to reduce cardiovascular risk, particularly in patients with coronary artery disease, heart failure, or CKD having concomitant hyperuricemia. Our large-scale study using population-based matched-cohort design in patients with gout and “normal risk” for cardiovascular events did not observe any beneficial effect of allopurinol, whereas the association with a modest increase in cardiovascular risk was detected. Several important risk factors for cardiovascular disease, such as smoking, alcohol consumption, body mass index, blood pressure were not obtainable in the current study, thus could potentially bias the effect estimate. A hypothesis-generating finding was suggested from a subgroup analysis of low- versus high-dose allopurinol therapy, showing a possible beneficial effect from high-dose therapy. Further prospective large-scale cohort studies or randomized controlled trials are needed.
